# Eight biomarkers on a novel strip for early diagnosis of acute myocardial infarction†

**DOI:** 10.1039/c9na00644c

**Published:** 2019-12-17

**Authors:** Li Huang, Yuanchang Zhang, Enben Su, Yuan Liu, Yan Deng, Lian Jin, Zhu Chen, Song Li, Yongxiang Zhao, Nongyue He

**Affiliations:** State Key Laboratory of Bioelectronics, School of Biological Science and Medical Engineering, Southeast University Nanjing 210096 China nyhe1958@163.com; Hunan Key Laboratory of Biomedical Nanomaterials and Devices, Hunan University of Technology Zhuzhou 412007 P. R. China; Getein Biotechnology Co., Ltd. Nanjing 210000 China; National Center for International Bio-targeting Theranostics, Guangxi Key Laboratory of Bio-targeting Theranostics, Collaborative Innovation Center for Targeting Tumor Theranostics, Guangxi Medical University Guangxi 530021 China yongxiang_zhao@126.com

## Abstract

Accurate detection of markers in human serum is important in the early diagnosis of acute myocardial infarction (AMI). This work presents a novel eight biomarker strip, which combines dry chemistry with a fluorescence lateral flow assay. Eight AMI markers were employed simultaneously for sensitive detection, including cholesterol (TC), triglyceride (TG), high density lipoprotein cholesterol (HDL-C) and low density lipoprotein cholesterol (LDL-C), uric acid (UA), myoglobin (Myo), creatine kinase-MB (CK-MB), and cardiac troponin I (cTnI). The strip offers the advantages of simple fabrication, convenience, time-saving detection and accurate assessment for AMI. Moreover, the strip possesses acceptable applicability for human serum. This proposed strategy establishes a remarkable platform for the construction of a multi-target detection strip that is feasible for accurate detection for real human serum samples.

## Abstract 2

Accurate detection of markers in human serum is important in the early diagnosis of acute myocardial infarction (AMI).

## Graphical abstract

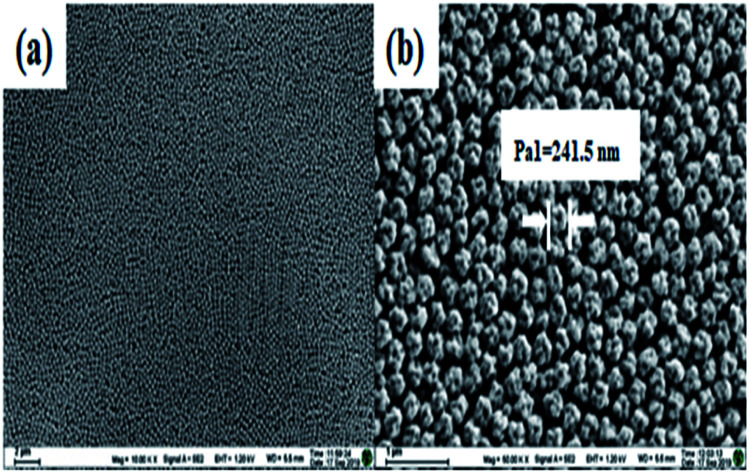

## Introduction

Recently, myocardial infarction has become a leading cause of death in most industrialized nations, especially in China.^1^ According to the 2013 China Cardiovascular Disease Report, deaths due to cardiovascular disease accounted for 38.7% of deaths in rural areas and 41.1% of deaths in urban areas. The prevention or treatment of acute myocardial infarction (AMI) in people is becoming especially urgent, while accurate diagnosis of patients is equally essential.^2^ According to the WHO guidelines, at least two out of the following three symptoms can be used to identify that a patient is at risk for AMI: characteristic chest pain, changes in the electrocardiogram (ECG) and elevation of the cardiac biomarkers in a serum sample.^3^

Comprehensive detection of the indexes of blood lipids including cholesterol (TC), triglyceride (TG), high-density lipoprotein cholesterol (HDL-C) and low-density lipoprotein cholesterol (LDL-C), uric acid (UA) and myocardial markers can be used for the monitoring and controlling of coronary heart disease, heart failure, and myocardial infarction.^4^ Up till now, there have been many approaches for the detection of serum lipids, UA and myocardial markers in clinics. Generally, TC and TG in serum are detected *via* chemical and enzymatic methods.^5^ Homogeneous methods, which include clearance and catalase clearance, the polyethylene glycol (PEG) modified enzyme method, and selective inhibition, are the recommended methods for HDL-C detection in clinics in China, and the LDL-C content in the serum sample is often calculated using Friedewald's formula (LDL-C = TC − HDL-C − TG/2.2 (unit: mmol dL^−1^) or LDL-C = TC − HDL-C − TG/5.0 (unit: mg dL^−1^)). However, it is noteworthy that the above formula is not universal. For example, if chylomicrons (CMs) exist in the detected serum, and the concentration of TG exceeds 4.52 mmol L^−1^ (400 mg dL^−1^) or the serum contains abnormal lipoproteins (hyperlipoproteinemia III), then the formula is not applicable.^6,7^ Moreover, there are several methods for UA detection, including ultraviolet spectrophotometry,^8^ the recombinant urate oxidase method,^9^ liquid chromatography,^10^ capillary ion analysis,^11^ electrochemical methods,^12^ the chemiluminescence flow injection method,^13^*etc.* However, they are mainly carried out on a large-scale blood biochemistry analyzer, which requires a large blood volume, and are often time-consuming and require expensive equipment.

Additionally, cardiac biomarkers including myoglobin (Myo), creatine kinase-MB (CK-MB), and cardiac troponin I (cTnI) are commonly tested for AMI assessment.^14^ The sensitive, specific and early detection of a definitive cardiac biomarker in the blood is essential for the proper treatment of AMI.^3,15,16^ There are many methods for the detection of markers of AMI, such as mass spectrometry,^17,18^ liquid chromatography,^19^ electrochemical analysis,^2,20^ Surface Plasmon Resonance (SPR),^21^ fluorescence methods,^22^ and colorimetric biosensing.^23^ Although most of the methods possess high sensitivity and selectivity, there still exist some limitations, such as expensive equipment, time consumption, and complicated expensive operation. Sensitive and convenient detection of AMI markers plays an important role in AMI detection, making them inapplicable in point-of-care (POC) testing.^24^ However, most of the detection methods are qualitative, with the concentration of the AMI markers being critical parameters for AMI assessment and prediction.^25,26^ In order to overcome these shortcomings, efforts have been made to develop simple, rapid, and efficient analytical techniques; hence, the necessity to develop a many-in-one detection strip for AMI detection is obvious.

**Scheme 1:**
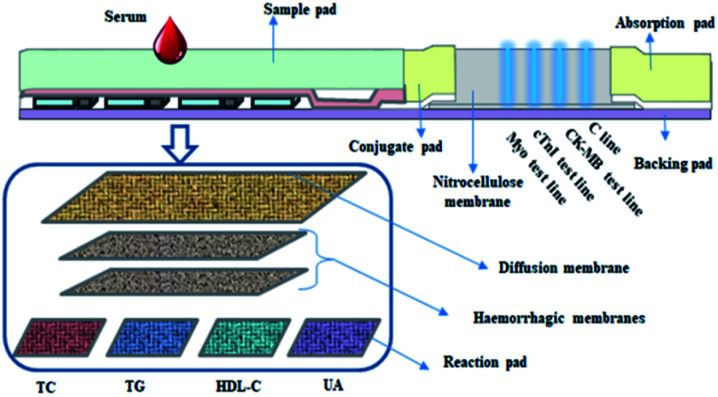
Schematic illustration of the eight biomarker strip for AMI.

Herein, we design and fabricate a novel dry chemistry-fluorescence lateral flow assay strip for AMI detection with Myo, CK-MB, and cTnI being detected by the fluorescence immunoassay approach, while TC, TG, HDL-C, and UA are detected *via* the dry-chemistry approach, with LDL-C being calculated. For AMI marker detection, monoclonal antibodies of Myo, cTnI, and CK-MB and rabbit anti-mouse polyclonal antibody were used. The concentration of the marker is reflected by the intensity. Blood lipids [TC, TG, HDL-C, and LDL-C (calculation)] and uric acid (UA) were detected based on the Trinder reaction, after which the analyzer reads the results by reflective photometry. According to regression analysis using a memory chip, the concentrations of TC, TG, HDL-C, UA and LDL-C were calculated. Hence, using the strip, eight biomarkers could be detected quantitatively at the same time with simple equipment exhibiting time-saving properties (Scheme 1).

## Experimental section

### Materials

Lipoprotein esterase, glycerol kinase, glycerol-3-phosphate oxidase, cholesterol oxidase, cholesterol esterase, urease, and peroxidase were provided by Japan Toyota Textile Company, while the dry chemistry reaction and filter membranes were provided by Pall Company, United States and diffusion membranes were provided by the Swiss company, Sefar. cTnI monoclonal antibody I (IC01) and monoclonal antibody II (1A4) and rabbit anti-mouse IgG antibody were provided by Getein Biotech, Inc., while cTnI monoclonal antibody III (7B9) and Myo monoclonal antibody II (MY03) were provided by Hytest Company and CK-MB monoclonal antibodies (M612 and M745), mouse-derived, were provided by Roche. Moreover, Myo monoclonal antibody I (MY02) was procured from Keyegg Biotechnology Co., Ltd., while nitrate fiber chromatographic (NC) membranes were purchased from Millipore Company. The reagents were purchased and used without further purification.

### Preparation of fluorescent latex microspheres

First, 12 mL of styrene and 2 mL of methacrylic acid were mixed with 200 mL distilled water by ultrasonication under a N_2_ atmosphere. Then 100 mL of divinylbenzene was added together with 200 mg potassium persulfate after heating to 75 °C. After reaction for 12 h the reaction temperature was adjusted to 30 °C. Then Bodipy 650 dissolved in dichloromethane was added and then the reaction was continued for 1 h. Finally, after removing dichloromethane (60 °C) with nitrogen and centrifugal cleaning, fluorescent microspheres were obtained. Then the fluorescent latex microspheres were labelled with cTnI, Myo, and CK-MB antibodies *via* the EDC/NHS method.

100 mL of the fluorescent microspheres (solid content is 1%), 500 mL of MES buffer (20 mM MES pH = 5.6) and 200 mL of 10 mg/mL EDC (20 mM MES pH 5.6) were allowed to react for 10 min, and then the mixture was centrifuged to remove the supernatant, and 200 mL of 10 mg/mL NHS (20 mM PB pH 7.2) was added, mixed for 10 min, and centrifuged at 14 000 rpm for 20 min. After removing the supernatant, 500 mL PB buffer (20 mM PB pH 7.2) was added to resuspend the fluorescent microspheres, 400 mL of 10 mg mL^−1^ CK-MB, cTnI and Myo antibody were added, respectively, allowed to react for 30 min, and centrifuged, and a preservation solution was used to resuspend the fluorescent microspheres (20 mM pH 7.2 PB + 0.1% BSA + 10% sucrose).

### Fabrication of the reaction membranes

The HDL test mother liquor, reaction liquor, and phosphotungstic acid treatment liquor were prepared (the detailed processes are explained in the ESI†). Then, the HDL reaction liquor was coated on the reaction membrane with a content of 1.0 μL cm^−1^, followed by drying at room temperature for 4–5 h. The same process was applied for UA, TG and TC reaction membranes. Then, the HDL and UA reaction membranes were immersed into 100 mL of phosphotungstic acid for 2 h, respectively, after which the membranes were dried at room temperature for 4–5 h.

### Fabrication of the myocardial triple detection membrane

The NC membrane was coated with a quality control line antibody and CK-MB, cTnI and Myo antibodies, respectively, followed by drying for 2–3 h at room temperature, to obtain the treated chromatographic membrane. Thereafter, the CK-MB, cTnI and Myo antibody latex microspheres were mixed in a ratio of 0.1 : 1 : 0.05 and sprayed on a fiberglass membrane at 1.5 μL cm^−1^. Finally, a fluorescent pad was obtained after drying for 2–3 h at room temperature.

### Fabrication of eight biomarkers in one detection kit for AMI detection

After the above-prepared reagent strips were completely dried, each item was assembled on the same eight-bar bottom plate, with the HDL reaction-, HDL treatment-, TG reaction-, TC reaction-, UA reaction-, blood filter- and diffusion membranes pasted on the bottom plate in sequence, while the sample and fluorescent pads, chromatographic membrane and absorbent paper were pasted in sequence in the myocardial triple detection area. Finally, it was evenly cut into narrow strips and assembled with a matching eight-bar shell, to obtain eight biomarkers on one detection strip for AMI detection.

### The mechanism of the fabricated strip

The eight biomarker strip was developed to quantitatively detect four blood lipids (TC, TG, HDL-C, and LDL-C), uric acid (UA), CK-MB, cTnI and Myo in human serum; 120 μL blood is required to test the strip. The data were collected using a Getein 3600 Biochemistry Immune Quantitative Analyzer (Getein Biotech, Inc, Nanjing, China) with a detection time of 3 min for the dry chemistry biomarkers and 10 min for the fluorescence lateral flow assay. The mechanism of the strip is as outlined: the dry chemical module reacts with the underlying reaction membrane, resulting in a color change, with the changing intensity of the color being related to the concentration of the analyte. The photoelectric signal is then inputted into the measuring system using a photoelectric scanning technique and the intensity of the photoelectric signal is analyzed, after which the concentration of the substance is obtained quantitatively. For the fluorescence lateral flow assay module, the measurement system automatically scans the labels and the binding area of the strip after reaction with the analytes to obtain optical signals which are then measured, and the concentration of the analytes is obtained quantitatively.

## Results and discussion

### Characterization of the fluorescent latex microspheres

**Fig. 1:**
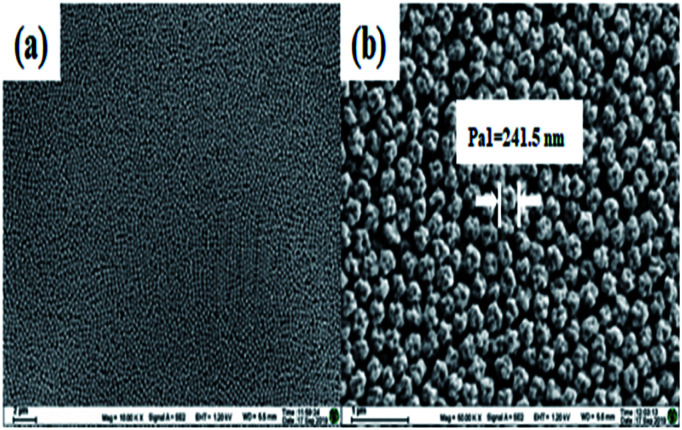
The SEM micrographs of the fluorescent latex microspheres at different magnifications. (a) ×10 000 and (b) ×50 000.

The fluorescent latex microspheres were prepared, and the morphology of the microspheres was characterized using a scanning electron microscope (SEM). As shown in Fig. 1, the microspheres are uniform in size (Fig. 1a) and the average diameter of the microspheres is 241.5 nm. Hence, the fluorescent latex microspheres provide a perfect platform for cTnI, Myo and CK-MB detection.

### Analytical performance of the eight biomarker strip

**Fig. 2:**
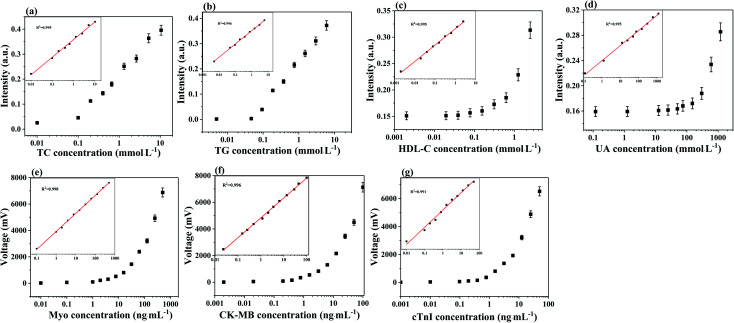
(a–g) The calibration curves of TC, TG, HDL-C, UA, CK-MB, cTnI and Myo. The error bars indicate the standard deviations calculated from three tests.

To analyze the performance of the eight biomarker strip based on the dry chemical analysis and immunofluorescence method, TC, TG, HDL-C, LDL-C, UA, CK-MB, cTnI and Myo in human serum samples were detected at the same time and the detection results are shown in Fig. 2. In order to evaluate the quantitative performance of the as-developed eight biomarker strip, we employed these strips for the detection of a series of samples with different concentrations of analytes for AMI, spiked into human serum (Table 1).

**Table 1:** Concentration of cardiac biomarkers

	TG (mmol L^−1^)	TC (mmol L^−1^)	HDL-C (mmol L^−1^)	UA (mmol L^−1^)	CK-MB (ng mL^−1^)	Myo (ng mL^−1^)	cTnI (ng mL^−1^)
1	6	10.5	2.50	1200	100	500	50
2	3	5.25	1.25	600	50	250	25
3	1.5	2.50	0.60	300	25	125	12.5
4	0.75	1.30	0.30	150	12.5	62.5	6.25
5	0.35	0.65	0.15	75	6.25	31.25	3.15
6	0.20	0.40	0.10	50	3.15	15.5	1.55
7	0.10	0.20	0.05	25	1.55	7.5	0.75
8	0.05	0.10	0.02	12.5	0.75	4	0.40
9	0.005	0.01	0.002	1.25	0.40	2	0.20
10	—	—	—	0.12	0.20	1	0.10
11	—	—	—	—	0.02	0.0	0.01
12	—	—	—	—	0.002	0.01	0.001

The concentrations were chosen to cover the clinical range of all the eight cardiac biomarkers in the human body. Taking TC as an example, the trend in intensity change with TC concentration is shown in Fig. 2a, while the consistency between the calculated and detected results is shown in the inset. Based on the TC detection results at different concentrations, the slope of the calibration curves for TC was determined to be *y* = 0.0511e^12.863*x*^. The quantitative results in Fig. 2a indicated a good linearity between the TC test results and the corresponding TC concentration (*R*^2^ = 0.995). Furthermore, the limit of detection (LOD) of TC is 0.01 mmol L^−1^. Similarly, the LOD values are 0.005 mmol L^−1^, 0.002 mmol L^−1^, 0.12 mmol L^−1^, 0.002 ng mL^−1^, 0.001 ng mL^−1^ and 0.01 ng mL^−1^ for TG, HDL-C, UA, CK-MB, cTnI and Myo, respectively (Fig. 2S†). Moreover, the consistencies between the calculated and detected results are acceptable (*R*^2^ > 0.994). The results of the eight biomarkers are of the same order of magnitude as the marketable products of Getein Biotech, Inc.

### Precision of the fabricated strips

The precision of the strip was assessed *via* the coefficient of variation (CV). As shown in Table 2, the CVs of TC, TG, HDL-C, UA, Myo, CK-MB, and cTnI are (4.4%, 2.4%), (4.2%, 1.80%), (5.00%, 2.50%), (5.1%, 1.80%), (4.69%, 4.92%), (5.80%, 4.53%), and (3.35%, 4.82%), respectively. The results showed that the strip possesses an acceptable precision for AMI detection and assessment.

**Table 2:** The CV results of TC, TG, HDL-C, UA, Myo, CK-MB, and cTnI

Analyst	TC		TG		HDL-C		UA		Myo		CK-MB		cTnI	
Detection range	2.90	7.24	0.97	4.68	0.29	1.63	78.00	751.00	80.70	514.00	7.41	76.85	0.32	45.65
CV (%)	4.40	2.40	4.20	1.80	5.00	2.50	5.10	1.80	4.69	4.92	5.80	4.53	3.35	4.82

### The specificity of the fabricated strips

**Fig. 3:**
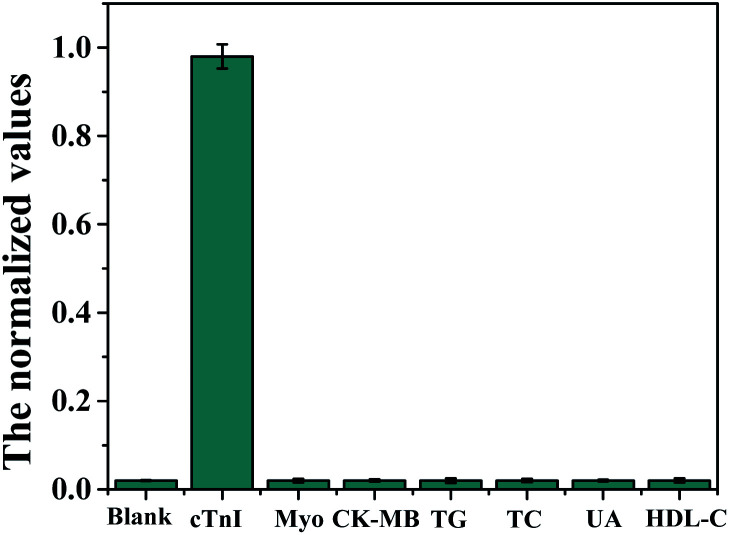
The selectivity of the prepared strips for cTnI detection. 50 ng mL^−1^ for cTnI and 500 ng mL^−1^, 500 ng mL^−1^, 50 mmol L^−1^, 50 mmol L^−1^, 50 mmol L^−1^, and 50 mmol L^−1^ for CK-MB, Myo, TC, TG, UA, and HDL-C, respectively, tested in three parallel measurements.

To demonstrate the specificity of the fabricated strips, cTnI was taken as an example of the analytes, and the other six analytes, including CK-MB, Myo, TC, TG, UA, and HDL-C were used as the negative controls. The concentrations of the background analytes were set to 500 ng mL^−1^, 500 ng mL^−1^, 50 mmol L^−1^, 50 mmol L^−1^, 50 mmol L^−1^, and 50 mmol L^−1^, respectively, for CK-MB, Myo, TC, TG, UA, and HDL-C. As shown in Fig. 3, cTnI exhibited significant signals, while the control analytes showed almost ignorable signals. Similar results for the other seven targets were obtained (not shown here). Therefore, the results demonstrated that the fabricated strips have good selectivity with cross-interferences and could be used in the detection of complex biological samples.

### Clinical sample analysis

To evaluate the clinical applicability and diagnostic capability of the fabricated eight biomarker strip, 30 serum samples collected from hospitalized patients suffering from AMI were analyzed with the strip and clinical methods (Beckman AU5800 automatic biochemistry analyzer and Abbott i2000SR chemiluminescence immunoassay (CLIA) analyzer, respectively). For the validation of the as-proposed assay technique, Passing–Bablok regression and Spearman's correlation coefficient were adopted to analyze the linear dependence between the two methods. This study was performed in strict accordance with The Technical Guidelines for *In Vitro* Diagnostic Reagents Clinical Trial (CFDA notice no. 16 2014) and was approved by the Ethics Committee of the Yangzhou First People's Hospital.

**Fig. 4:**
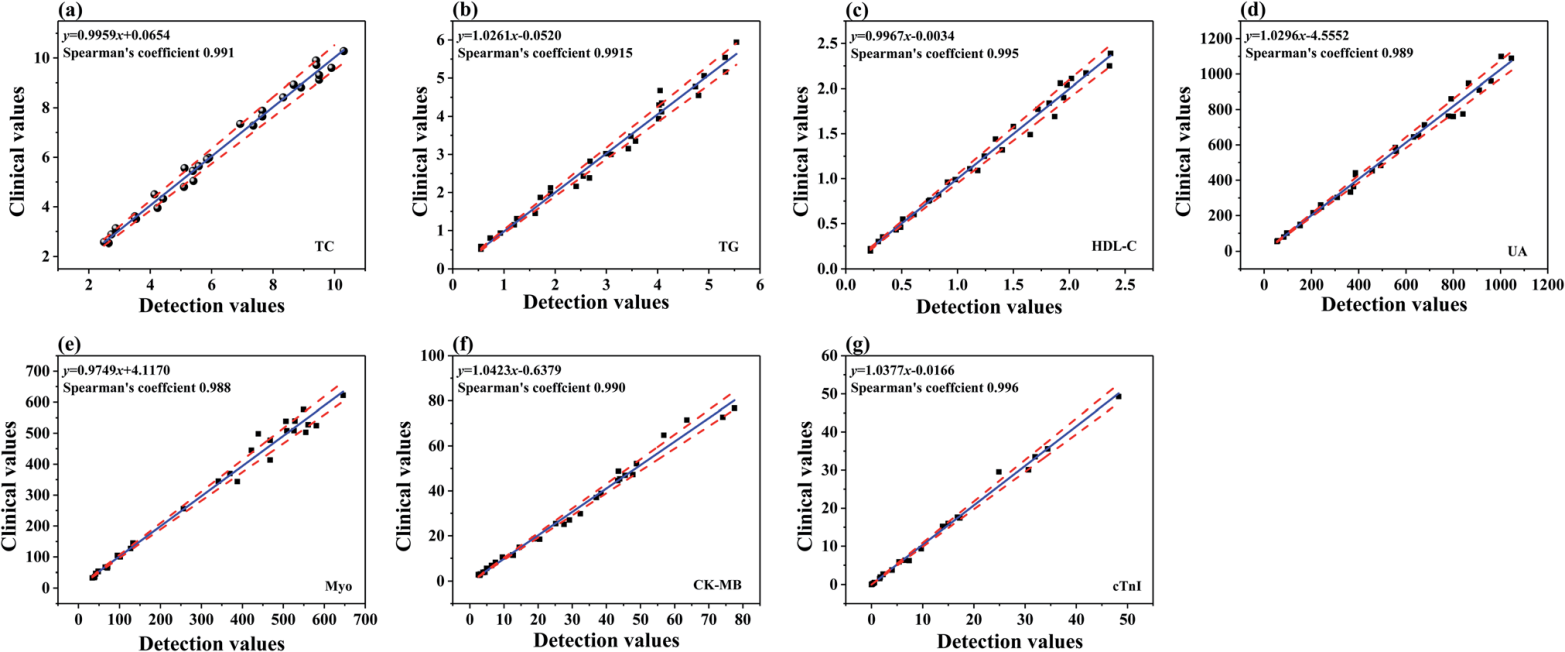
Passing–Bablok regression lines between the results obtained from clinical methods and the strip for TC, TG, HDL-C, UA, Myo, CK-MB and cTnI (a–g) detection in real serum samples (in the figures, the solid lines represent the linear regression line, with the dashed lines showing the range of 95% CI).

The results are shown in Fig. 4a–g, while the regression equation and Spearman's correlation coefficients of rank correlation of TG, TC, HDL-C, LDL-C, and UA, between clinical values (AU5800) and strip detection values, and Myo, cTnI, and CK-MB, between clinical values (CLIA) and strip detection values, are all shown in the inset. Both the slopes of the eight regression equations and the Spearman correlation coefficients are close to 1, which are included in the 95% confidence intervals (CIs) of the corresponding parameters. This indicated that the results of the eight biomarker strip and clinical values for the eight markers of AMI detection possess good linear correlation. Obtaining the conventional clinical values required sample pretreatment (about 1 h), expensive instruments and trained personnel for the assays, but in contrast, the novel eight biomarker strip is free of sample pretreatment in clinical applications, low-cost, easy to use, and more sensitive, and the reaction result is ready to be read in 15 min, which is faster and more convenient than when using the big analyzer. Therefore, the fabricated strip is more favorable for precision AMI diagnosis and prognosis in the hospital, which will greatly save time for AMI patients, in addition to lowering the risks of AMI.

## Conclusions

In summary, an eight biomarker strip based on dry chemical analysis for TG, TA, HDL, LDL, and UA detection and immunofluorescence for Myo, CK-MB, and cTnI quantitative detection was fabricated. The LOD results for the analytes revealed the same order of magnitude as that of the market strip with negligible cross-interferences. Moreover, the CV was used for assessing the precision of the strip, with the results showing that the strip exhibits acceptable precision for AMI detection and assessment. Finally, the fabricated strips were employed for clinical detection in real human serum and the obtained results of the strip and clinical methods (automatic biochemistry analyzer and CLIA) for the detection of eight markers of AMI possess good linear correlation. Therefore, the strip is more favorable due to its precision of AMI diagnosis and prognosis in hospitals, which will not only greatly save time for AMI patients, but also lower the risks of AMI.

## Conflicts of interest

There are no conflicts to declare.

## Supplementary Material

NA-002-C9NA00644C-s001
