# Supplementary figures and images for: Eight biomarkers on a novel strip for early diagnosis of acute myocardial infarction
Source: Nanoscale Adv. 2019 Dec 17;2(3):1138–43. doi: 10.1039/c9na00644c (PMC9419248; doi:10.1039/c9na00644c)

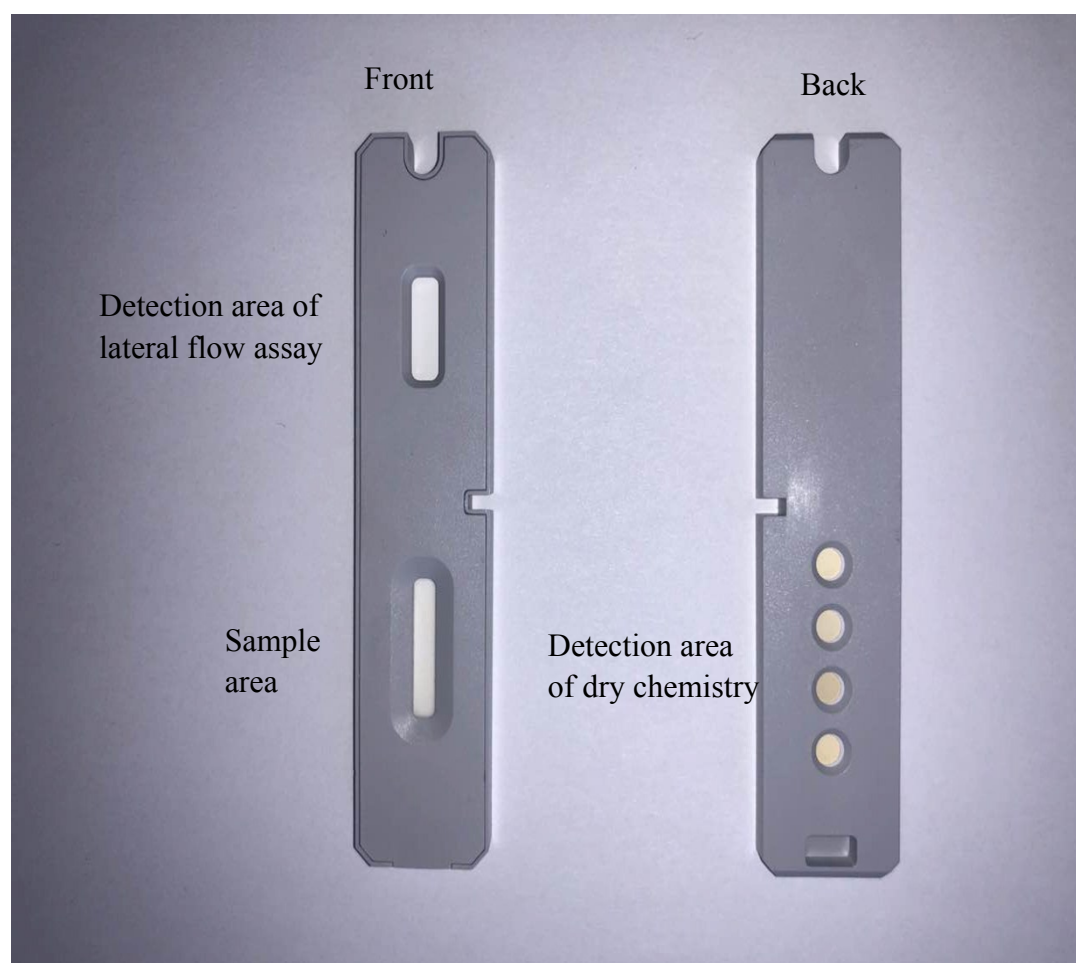

Figure S1 Actual picture of reagent strip

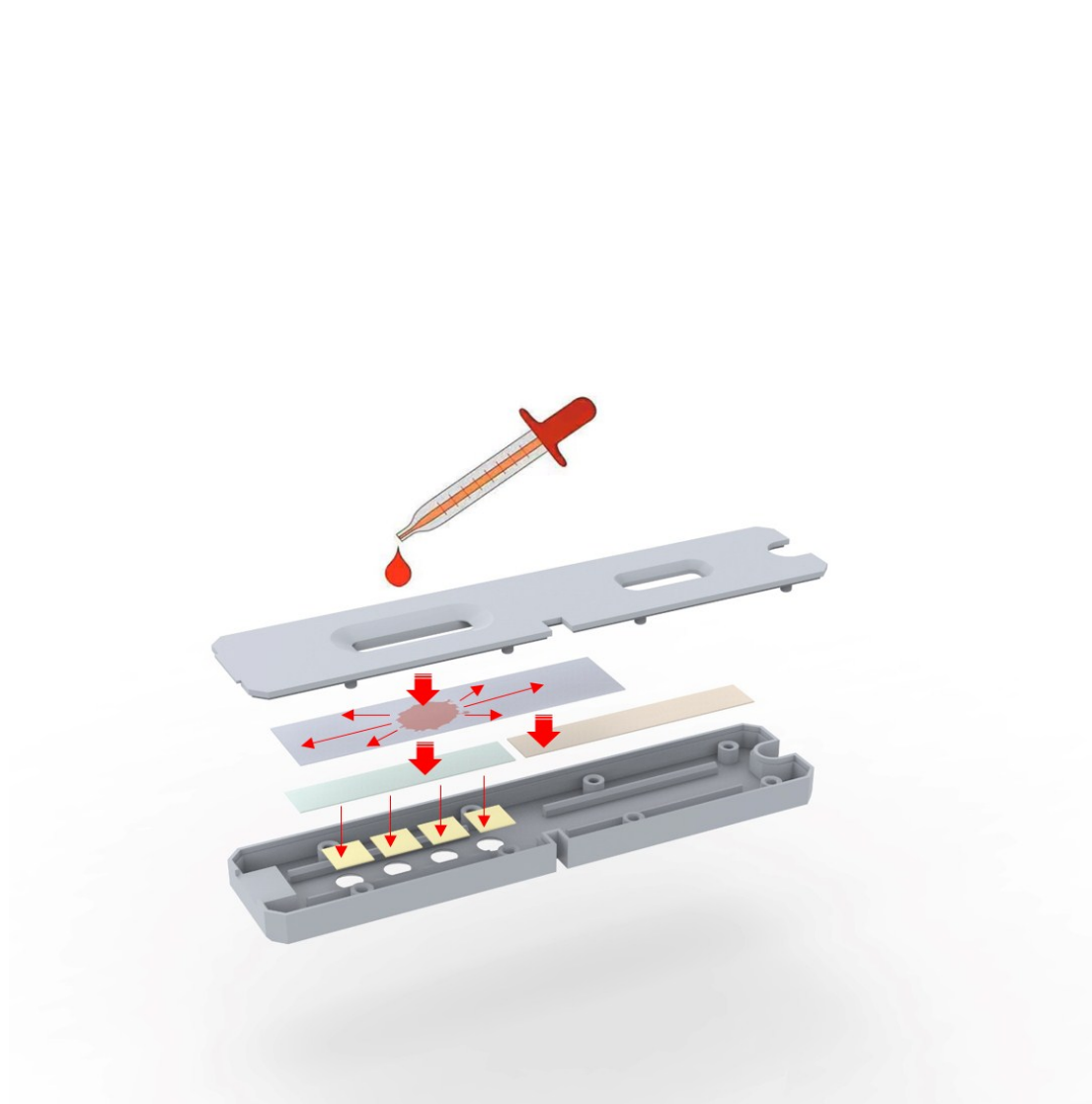

Figure S2 Schematic drawing of the strip device

Supplement: NA-002-C9NA00644C-s001 [file NA-002-C9NA00644C-s001.pdf]
